# Characterization and anti-inflammatory studies of supramolecular assemblies of chlorogenic acids with metal ions

**DOI:** 10.3389/fphar.2025.1726226

**Published:** 2026-02-09

**Authors:** Zhen-Min Zhang, Ru-Yue Zhang, Lian Zhu, Zi-Xuan Liu, Han-Xiu Deng, Jie-Fu Tang, Jia-Yu Zhang, Wei Cai

**Affiliations:** 1 School of Pharmacy, Binzhou Medical University, Yantai, Shandong, China; 2 School of Pharmacy, Hunan University of Medicine, Huaihua, Hunan, China; 3 The First Affiliated Hospital of Hunan University of Medicine, Huaihua, Hunan, China

**Keywords:** anti-inflammation, chlorogenic acids, metal ions, NF-κB signaling pathway, supramolecular assemblies

## Abstract

**Introduction:**

Self-assembled natural small molecules are believed to have numerous potential applications, particularly in the material and pharmaceutical industries, as self-assembly increases the anti-inflammatory, antitumor, antiviral, and other biological activities. In this study, we synthesized more potent potential anti-inflammatory agents by combining chlorogenic acids with metals (iron and copper).

**Methods:**

Supramolecular assemblies were synthesized by combining chlorogenic acids with iron and copper. The synthesis conditions were optimized using mass spectrometry. The resulting complexes were comprehensively characterized by ultraviolet-visible spectroscopy, Fourier-transform infrared spectroscopy, and mass spectrometry to confirm their formation and stoichiometry. The anti-inflammatory activity was evaluated in vitro using lipopolysaccharide-induced RAW264.7 macrophages. The production of inflammatory mediators (NO, IL-6, IL-1β, TNF-α) was measured. Mechanistic studies were conducted to assess the effects on the NF-κB signaling pathway and the expression of downstream proteins iNOS and COX-2.

**Results:**

Characterization data confirmed the successful formation of chlorogenic acid-metal supramolecules, primarily in a 1:1 stoichiometry. These metal-based complexes exhibited significantly enhanced anti-inflammatory effects compared to the parent chlorogenic acid molecules. *In vitro* assays demonstrated their potent suppression of key inflammatory mediators, including NO, IL-6, IL-1β, and TNF-α. Mechanistic investigations revealed that the enhanced activity was achieved through the effective inhibition of the NF-κB signaling pathway, leading to the downregulated expression of iNOS and COX-2 proteins.

**Discussion:**

The findings confirm that complexation with metals (iron and copper) successfully enhances the anti-inflammatory efficacy of chlorogenic acids. The primary mechanism involves the inhibition of the NF-κB pathway. This study provides a novel and efficient strategy for augmenting the bioactivities of natural products and highlights the considerable potential of chlorogenic acid-metal supramolecular assemblies as a promising new class of anti-inflammatory agents.

## Highlights

- Self-assembly of natural small-molecule chlorogenic acids with iron and copper was investigated.
- Anti-inflammatory effects and action mechanisms of neochlorogenic acid supramolecules were explored.
- Chlorogenic-acid–metal supramolecules are proposed as a new class of anti-inflammatory agents.

## 1 Introduction

Supramolecular structures refer to the spontaneous assembly and association of molecules of the same or different species through various non-covalent interaction forces to form multimolecular aggregates (Lehn, 2002).

Historically, natural products like small molecules derived from plants and animals (Zhou et al., 2022) have been regarded as the largest source of drug lead compounds owing to their diverse structures and broad pharmacological activities (Chen and Ding, 2023; Xu et al., 2021). At present, the utilization of natural small molecules and their derivatives to develop new drugs is considered an effective measure for preventing and treating various diseases (Chen et al., 2024; Gan et al., 2024) given the increasing numbers of studies on the self-assembly of natural small molecules as well as their applications (Hou et al., 2022; Ji et al., 2022; Yong, 2024; Yang et al., 2023; Yuan et al., 2021). By analyzing the structural formulas and comparing known self-assembled natural small molecules (Xiang et al., 2024), we found that there are greater or fewer numbers of olefin groups in these structures, such as C=O and C=C bonds, implying that the π–π packing forces play important roles in the molecular assembly (Wang et al., 2024; Yong, 2024). In particular, in molecules containing the benzene ring structure, expansion of the π electron conjugation causes obvious π–π accumulation between the aromatic rings, which significantly impacts the spatial arrangement of the molecules (Umadevi et al., 2014).

Natural small-molecule chlorogenic acids (CGAs) are commonly found to occur naturally in plants (Barcellos Silva et al., 2024). Importantly, CGAs are an important type of bioactive dietary polyphenol that has many therapeutic effects, such as antiobesity, hypoglycemic, antioxidant, antibacterial, anti-inflammatory, neuroprotective, antiviral, antibacterial, anti-cardiovascular disease, and immune regulation activities (Fu et al., 2016; Padilla et al., 2022; Xi et al., 2022; Zhao et al., 2022). The CGA structure comprises C=C, C=O, and benzene ring components, which have relatively large conjugated systems and can easily form supramolecules with metal ions that are detectable by ultraviolet-visual (UV-vis) spectroscopy, Fourier-transform infrared (FT-IR) spectroscopy, and mass spectrometry (MS) (Luo and Xia, 2021).

Inflammation is a predominant sign that is observed in all phases of disease. The important inflammatory response mechanisms are represented by the molecular synergy between induced nitric oxide synthase (iNOS) and cyclooxygenase-2 (COX-2) (Kim et al., 2005). The expression of both COX-2 and iNOS is regulated by nuclear factor kappa B (NF-κB), and the presence of p50/p65 in the nucleus and additional binding of p65 at the COX-2 promoter region most likely contribute to the expression of COX-2 (Mortezaee, 2018; Pasha et al., 2024), The NF-κB signaling pathway induces the expression of target genes like *IL-6*, *TNF-α*, and *IL-1β* (Fakhri et al., 2021) while also playing vital roles in many conditions like immune and inflammatory responses, cell growth, viral infections, and ulcerative colitis (Kumar et al., 2004).

The therapeutic potentials of supramolecular assemblies, particularly those derived from natural products, have garnered significant interest in recent years owing to their ability to enhance drug bioavailability, stability, and efficacy (Sun et al., 2021). For instance, supramolecular hydrogels constructed from natural small molecules have been demonstrated as promising functional biomaterials for drug delivery and tissue regeneration (Huang et al., 2022). Notably, a recent study demonstrated the self-assembly of CGAs into a hydrogel capable of accelerating wound healing, highlighting the intrinsic propensity for self-assembly and application potential of CGAs (Huang et al., 2023). Beyond organic self-assembly, the coordination of phenolic acids with metal ions to form functional complexes represents another compelling strategy. Previous research efforts have primarily focused on the antioxidant and antimicrobial properties of such metal–phenolic complexes. However, a systematic investigation into the deliberate design of chlorogenic-acid–metal supramolecular assemblies for specifically enhancing anti-inflammatory activity along with an in-depth exploration of the underlying cellular mechanisms remains largely unexplored.

In the present study, we synthesized and characterized a new class of supramolecules comprising CGAs and metal ions. Furthermore, we conducted a series of *in vitro* experiments to investigate the efficacies of these complexes to determine if they can be used as more effective anti-inflammatory agents. We also studied the supramolecular anti-inflammatory mechanisms based on interactions with the NF-κB/COX-2/iNOS pathway.

## 2 Experimental procedures

### 2.1 Materials

Neochlorogenic acid (NA), cryptochlorogenic acid (CCA), and isochlorogenic acid A (ICAA) were purchased from Chengdu Herpurify Co., Ltd.; the purities of all three CGAs were above 98%. Acetonitrile (ACN) and methanol of liquid chromatography mass spectrometry (LC-MS) grade were purchased from Fisher Scientific, Inc. We used deionized water in the experiments, and all remaining reagents were analytical grade. Lastly, FeCl_3_·6H_2_O and CuCl_2_·2H_2_O were purchased from Kermel; N-omega-nitro-L-arginine methyl ester (L-NAME) was purchased from APExBIO Technology LLC; the Griess reagents and cell counting kit-8 (CCK-8) were purchased from Beyotime; the lipopolysaccharide (LPS) and enzyme-linked immunosorbent assay (ELISA) kits were purchased from Thermo Fisher Scientific.

### 2.2 Preparation of supramolecules

Approximately 7.08 mg of the CGAs and 5.40 mg of FeCl_3_·6H_2_O or 6.8 mg of CuCl_2_·2H_2_O were weighed and mixed with 20 mL of ultrapure water; this mixture was refluxed for 2 h at 60 °C.

Optimization of the synthesize processes of supramolecules by single-factor experiments

Accordingly, three single factors were investigated, namely, reaction temperature, molar ratio of the origin drug with metal, and reaction time, for the relative abundances of the supramolecules and solution states. Each of the three single factors was further explored at five levels as follows: reaction temperatures of 30, 45, 60, 75, and 90 °C; molar ratios of origin drug and metal at 3:1, 2:1, 1:1, 1:2, and 1:3; reaction times of 1, 1.5, 2, 2.5, and 3 h.

### 2.3 Characterization using UV-vis spectroscopy

The spectral characteristics of the CGAs and their supramolecules were clarified by UV-vis spectroscopic techniques. The UV spectra of the supramolecules were resolved using a Shimadzu UV2600 UV-vis spectrophotometer (Kobe, Japan) in the wavelength scanning range of 200–500 nm.

### 2.4 Characterization by FT-IR spectroscopy

The supramolecules were lyophilized, ground with potassium bromide, and squeezed into tablets. Then, their infrared spectra were obtained with a spectral resolution of 4 cm^–1^ using a Shimadzu IRAffinity-1S spectrophotometer (Kobe, Japan). At the start of the measurements, a spectrum representing the background was obtained.

### 2.5 Characterization by MS

#### 2.5.1 LC-MS

A Dionex Ultimate 3,000 ultrahigh-performance liquid chromatography (UHPLC) system (Dionex, Inc., United States) was used for the analysis. The separations were performed on an ACQUITY Premier BEH C18 (2.1 mm × 100 mm, 1.7 μm) column. A 14 min gradient of ACN (A) and 0.1% formic acid water (B) was employed at a flow rate of 0.28 mL min^–1^. Then, a linear gradient with the following proportions (v/v) of solvent B was applied: 0–1 min, 92% B; 1–7 min, 92%–80% B; 7–10.5 min, 80%–60% B; 10.5–10.6 min, 60%–5% B; 10.6–12 min, 5% B; 12–12.1 min, 5%−92% B; 12.1–14 min, 92% B. The column temperature was maintained at 40 °C, and a 2-μL sample was injected with a spray voltage of 3.5 kV, sheath gas flow rate of 35 arbitrary units, and auxiliary gas flow rate of 10 arbitrary units. The capillary temperature and auxiliary gas heater temperature were 320 °C and 350 °C, respectively, while the S-lens RF level was 60, orbitrap resolution was 70,000 full-width at scanning range, and collision energy was 35 eV.

#### 2.5.2 Infusion

The molecular weights of the supramolecules were analyzed using the UHPLC-Q-Exactive Orbitrap MS/MS system. The electrospray ionization mode was considered positive (ESI+) with a capillary voltage of 3 kV, capillary temperature of 300 °C, auxiliary gas flow rate of 10 μL min^–1^, auxiliary gas heater temperature of 100 °C, and sheath gas flow rate of 15 arbitrary units. The mass-to-charge ratio (*m/z*) was determined to range from 80 to 1,200.

#### 2.5.3 Mass spectrometry imaging (MSI)

The MSI experiments were performed with an ambient air-flow-assisted desorption electrospray ionization (AFADESI) platform equipped with the Q-Exactive Orbitrap MS/MS system.

The sample surface was continuously scanned in the *x* direction at a constant rate of 0.2 mm s^–1^ for the AFADESI-MSI analysis and was separated by a vertical step of 0.2 mm in the *y* direction. For a mass resolution of 70,000 and scanning range of 80–1,200 Da, the mass spectra were obtained in positive full MS mode; the spray voltage was set to 5 kV, capillary temperature was 350 °C, and nitrogen (0.5 MPa) and methanol/water (75:25, v/v, 5 μL min^–1^) were used as the spray gas and spray solvent, respectively.

### 2.6 CCK-8 assay

The CCK-8 test was employed to quantify the cytotoxicity of the supramolecules. Accordingly, 2 × 10^4^ RAW264.7 cells (FuHeng Biology, Shanghai, China) were seeded per well in 96-well plates using complete media. The cells were cultured for 24 h at 37 °C in a CO_2_ incubator. Next, various doses (25, 50, and 100 μg mL^–1^) of the supramolecular compounds were applied to the cells; after 2 h, the cells were stimulated with LPS (500 ng mL^–1^) for 18 h. During the assay, CCK-8 reagent was added to each well, and the cells were left in the dark for one hour; then the optical density (OD) was obtained at 450 nm using the SPECTROstar Nano Microplate Reader (BMG Labtech, Ortenberg, Germany).

### 2.7 Determination of NO levels

The RAW264.7 cells were seeded at 5 × 10^4^ cells per well on a 96-well culture plate for a duration of 24 h; then, the cells were pretreated with a combination of the supramolecules or L-NAME for 2 h prior to being stimulated with LPS (500 ng mL^–1^) for 18 h in Dulbecco’s modified eagle medium (DMEM). The concentration of NO (1–100 μM) in each culture was detected using the Griess reagent, and the OD value was measured at 540 nm using a microplate reader.

### 2.8 ELISA

The anti-inflammatory activities of the synthesized compounds were evaluated by inhibiting the release of TNF-α, IL-6, and IL-1β from the LPS-induced RAW264.7 cells. Accordingly, the cells were first incubated for 24 h, and NA was applied for 2 h, followed by 18 h of stimulation with LPS. To measure the levels of TNF-α, IL-6, and IL-1β in the medium, the supernatant was collected and tested using an ELISA kit. The absorbance (450 nm) was measured using the SPECTROstar Nano Microplate Reader and interpolated using the standard curve.

### 2.9 Western blot analysis

The RAW264.7 cells were seeded in a 60-cm dish at a density of 2 × 10^6^ cells and stimulated with LPS (500 ng mL^–1^) along with NA, neochlorogenic-acid–iron supramolecular complex (NA–Fe), and neochlorogenic-acid–copper supramolecular complex (NA–Cu) (25, 50, and 100 μg mL^–1^) for 18 h. Then, the culture medium was discarded and the cells were washed twice with chilled phosphate-buffered saline (PBS); next, we added RIPA lysis buffer with 1% phenylmethylsulfonyl fluoride solution and 2% protease and phosphatase inhibitor (Beyotime, China) to the lysate to collect the cells before centrifuging them for 20 min at 4 °C after 20 min of lysis on ice. The protein grouping of each sample was measured utilizing the BCA protein assay kit (Beyotime, China) and separated by SDS-PAGE electrophoresis before being transferred to a polyvinylidene fluoride membrane and blocked for 1 h. The samples were incubated overnight at 4 °C with the following primary antibodies: COX-2, iNOS (Cell Signaling Technology, Danvers, MA, United States), NF-κB p65, phospho-NF-κB p65 (Immunoway, United States), and β-actin (Proteintech, China). The membranes were washed with Tris-buffered saline containing Tween 20, incubated with horseradish-peroxidase-conjugated secondary antibody for 1 h at room temperature, washed again, and developed after chemiluminescence.

### 2.10 Immunofluorescence analysis

To detect iNOS expression, the cells were cultured on a 24-well plate for 24 h, followed by treatment with NA, NA–Fe, and NA–Cu, and finally stimulated with 500 ng mL^–1^ of LPS for 18 h. To identify iNOS expression, the cells were fixed with 4% paraformaldehyde, permeabilized with 0.1% Triton X-100, and blocked with 5% bovine serum albumin. The iNOS antibody was incubated sequentially with the cells overnight at 4 °C and Alexa Fluor 488-labeled goat anti-rabbit IgG (H + L) for 1 h at room temperature, followed by washing with PBS buffer. All images were captured using a ZEISS LSM 980 device with Airyscan 2 (Carl Zeiss Inc., Oberkochen, Germany).

### 2.11 Statistical analysis

All data were represented as mean ± standard deviation (n = 3), and the differences among groups were assessed by one-way ANOVA using GraphPad Prism 7 software. A *p*-value <0.05 was considered to indicate a statistically significant difference.

## 3 Results and discussion

### 3.1 UV-vis spectra

**FIGURE 1:**
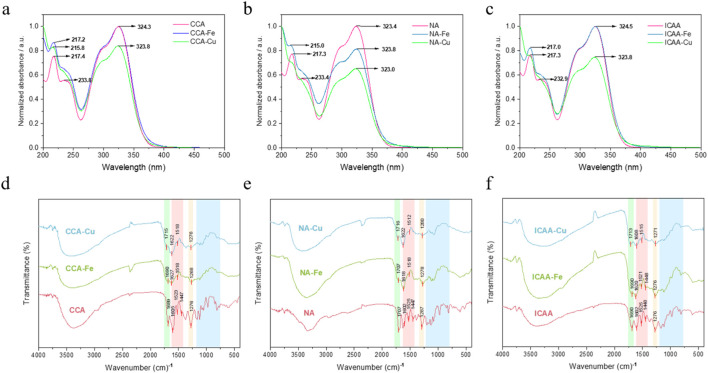
**(a–c)** Ultraviolet and **(d–f)** Fourier-transform infrared spectra of different chlorogenic acids and their supramolecules. CCA, cryptochlorogenic acid; NA, neochlorogenic acid; ICAA, isochlorogenic acid A; X–Fe, chlorogenic-acid–iron supramolecular complex; X–Cu, chlorogenic-acid–copper supramolecular complex.

In the UV range, all origin drugs (CCA, NA, and ICAA) showed a maximum absorption peak at 323 nm (Adnan et al., 2020), while the supramolecules demonstrated a faint red-shift accompanied by a change in the peak intensity. There was another stronger absorption peak at 217 nm in the origin drugs in this band; a blue-shift was observed in the Fe-based supramolecules, while the Cu-based supramolecules showed only faint absorption. All of these changes are attributable to the colloidal aggregation induced by ligand adsorption (Biswas et al., 2007), which changes the environment of the chromophore and prompts the intensity and red/blue shifts of the absorption peak (Figures 1a–c).

### 3.2 FT-IR spectra

The representative FT-IR spectra for the different CGAs and their supramolecular complexes are shown in Figures 1d–f, where several prominent bands are observed in the wavenumber range of 1,700–800 cm^–1^. The bands distinguishable at 1,447 cm^–1^, 1,523 cm^–1^, 1,605 cm^–1^, and 1,689 cm^–1^ are the characteristic bands of CGAs (Liang et al., 2016).

The bending of the C=O carbonyl group resulted in the band at 1,689 cm^–1^, while the phenyl CH shaking vibrations are expected to cause the band at 1,276 cm^–1^; moreover, the infrared absorption bands at 1,605 cm^–1^, 1,523 cm^–1^, and 1,447 cm^–1^ are assigned to stretching of the phenyl ring.

In contrast, the FT-IR spectra of the supramolecules exhibit significant alterations. Although most absorption features are retained, several characteristic peaks in the 800–1,700 cm^–1^ region either vanish or show notable red-shifts. These spectral changes, particularly the shifting and weakening of the carbonyl stretching band, strongly suggest the involvement of carbonyl and phenolic oxygen atoms in coordination with metal ions, confirming successful formation of the supramolecular complexes.

### 3.3 MS detection

**FIGURE 2:**
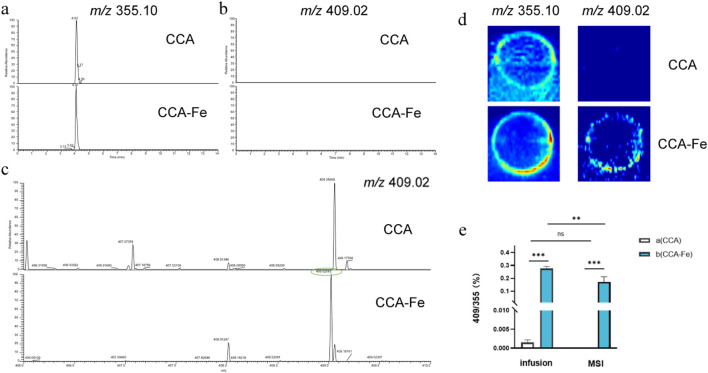
Extracted ion chromatograms of the three injection modes: **(a, b)** ultraperformance liquid chromatography, **(c)** infusion, and **(d)** mass spectrometry imaging. **(e)** Comparison of infusion and mass spectrometry imaging injection.

In these experiments, MSI as well as two injection modalities of the ESI-MS assay, namely, infusion and UPLC injection, were investigated. As shown in Figures 2a–d, only CGAs are detected when the reaction supernatant was injected with UPLC, possibly owing to the unstable decompositions of the supramolecules or dead adsorption on the column. During MS, the unreacted CCA (*m/z* = 355.10) as well as compounds with *m/z* values of 409.02, 408.01 (Fe), 416.01, and 417.02 (Cu) were detected in the reaction solution. At the same time, the adduct ions of CGA are generated, which are consistent with the precursor ion peaks of the supramolecules; however, their abundances are much lower than those of the supramolecules, such that the supramolecules can be identified by the ratio of the precursor ion peak of the CGA (*m/z* = 355). By comparing the two injection modes of infusion and MSI, it was found that infusion was more suitable for detection owing to the issue of extraction efficiency, as shown in Figure 2e. After a simple optimization of the MS conditions, the samples were retested under the infusion mode with the following conditions: flow rate of 10 μL min^–1^, auxiliary gas flow rate of 35 arbitrary units, capillary temperature of 300 °C, and spray voltage of 3 kV.

### 3.4 Single-factor experiments

Since the detected supramolecules are mainly composed of one molecule each of CGA and metal, we conducted single-factor experiments to control the synthesis conditions to produce more supramolecules. The ratio of the precursor ion peaks of the supramolecule to CGA was used as the index to represent the changes in the number of supramolecules.

#### 3.4.1 Effect of molar ratio of origin drug to metal

**FIGURE 3:**
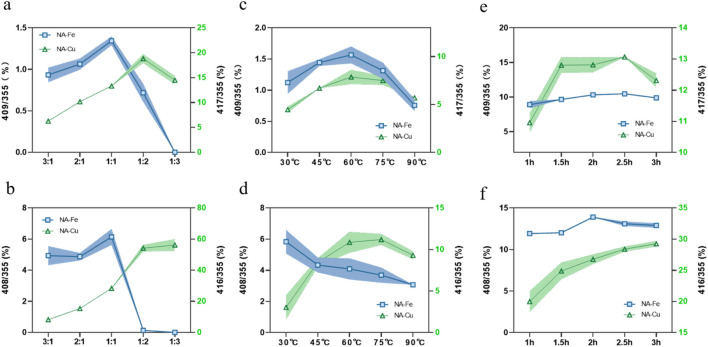
Influences of **(a, b)** molar ratio of the origin drug to metal, **(c, d)** reaction temperature, and **(e, f)** reaction time on the supramolecular complexes.

The parent ion peak pattern changes with an increase in the molar ratio of the parent drug, as shown in Figures 3a,b; as the molar ratio increases, the *m/z* value first increases and then trends downward, reaching a ratio of 1:1/1:2 before decreasing. The reason for this pattern may be that each CGA molecule is attached to only one metal molecule in the early stage before the *m/z* ratio increases. However, when there are more metals, more than one CGA molecule is connected to each metal molecule, such that the *m/z* ratio decreases. Hence, 1:1 (Fe) and 1:2 (Cu) were selected as the molar ratios for the follow-up tests.

#### 3.4.2 Effect of reaction temperature

It can be seen from Figures 3c,d that the reaction temperature has a significant impact on the supramolecule. Very high or very low temperatures result in diminished *m/z* values; such decreases are particularly evident at temperatures above 75 °C. The results show that high and low temperatures are not suitable, so 60 °C was chosen as the optimal temperature.

#### 3.4.3 Effect of reaction time

The experiments show that the *m/z* ratio increases with reaction time; however, when the reaction time exceeded a certain value, the increase in *m/z* was difficult to observe. Thus, considering the actual operations, 2 h (Fe) and 2.5 h (Cu) were selected as the best durations for synthesizing the corresponding supramolecules (Figures 3e,f).

### 3.5 Evaluation of anti-inflammatory activities

#### 3.5.1 Cytotoxicity assessment

**FIGURE 4:**
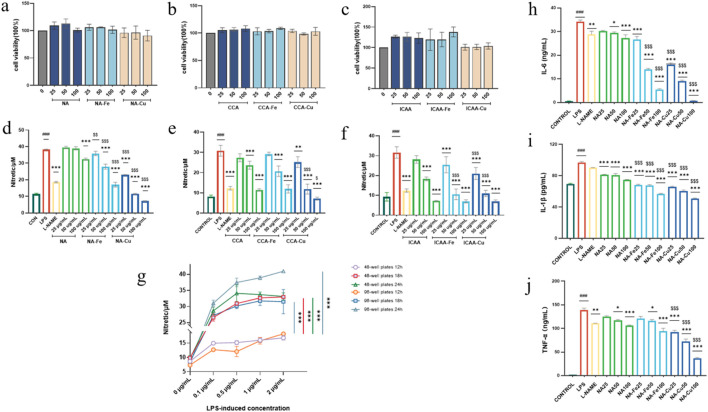
**(a–c)** Cytotoxicity analysis of different chlorogenic-acid (CGA)-based drugs and their supramolecular complexes on RAW264.7 cells. **(d–f)** Inhibitory effects of different CGA-based drugs and their supramolecular complexes on the production of NO. **(g)** Inflammation induction using different concentrations (0, 0.1, 0.5, 1, and 2 μg mL^–1^) of lipopolysaccharide (LPS) on RAW264.7 cells seeded in 96-well and 48-well plates for 12, 18, and 24 h. **(h–j)** Ability of neochlorogenic acid (NA) and it supramolecular complexes (NA–Fe and NA–Cu) applied at different concentrations to inhibit inflammatory cytokines IL-6, TNF-α, and IL-1β, respectively. The data are presented as mean ± standard deviation (n = 3); “*” compared to the LPS group, *p < 0.05, **p < 0.01, ***p < 0.001; “$” compared to the chlorogenic acids group, ^$^ *p* < 0.05, ^$$^ *p* < 0.01, ^$$$^ *p* < 0.001; “#” compared to the control group, ^###^ *p* < 0.001.

The cytotoxicities of the compounds were evaluated against RAW264.7 cells to guarantee safety during the anti-inflammatory assessments, so all prepared compounds were initially incubated for 24 h. As shown in Figures 4a–c, the prepared compounds did not have any negative effects on the cells and did not significantly encourage cell proliferation except for ICAA and its Fe-based supramolecules.

#### 3.5.2 Inhibition of inflammatory factor NO

As shown in Figures 4d–f, the prepared compounds were evaluated for hindering overgenerated NO in LPS-actuated RAW264.7 cells, where L-NAME was used as the positive control. To establish an optimal inflammatory model, the RAW264.7 cells were first seeded in both 96-well and 48-well plates and stimulated with LPS in a range of concentrations (0, 0.1, 0.5, 1, and 2 μg mL^–1^) for 12, 18, and 24 h. The results showed that stimulation with 0.5 μg mL^–1^ of LPS for 18 h in the 96-well plate format induced a robust and significant increase in NO production. Based on these observations and considering the higher throughput of the 96-well plate for drug screening, we selected the 0.5 μg mL^–1^ concentration of LPS with 18-h induction as the standard condition for all follow-up anti-inflammatory tests.

All compounds showed dose-dependent inhibition of NO production upon induction with LPS. We selected NA and its supramolecules for the subsequent bioevaluations owing to their superior inhibitory activities against NO production and low cytotoxicity on the LPS-induced RAW264.7 cells.

#### 3.5.3 Production of inflammatory cytokines

NA, NA–Fe, and NA–Cu were observed to inhibit the release of inflammatory factors IL-6, IL-1β, and TNF-α in RAW264.7 cells stimulated with LPS based on ELISA evaluations (Figure 4g). Figures 4h–j show that LPS-stimulated cells produced significantly higher amounts of inflammatory cytokines than untreated cells. Additionally, L-NAME successfully inhibited LPS-induced production of inflammatory factors. In the concentration range of 25–100 μg mL^–1^, NA was observed to diminish the production of inflammatory factors in a dose-dependent manner. Administration of NA-Fe and NA-Cu to RAW264.7 cells showed lower production of inflammatory cytokines compared to application of NA in the concentration range of 25–100 μg mL^–1^.

#### 3.5.4 Effects of NA and its supramolecules on activation of the NF-κB signaling pathway

NF-κB is known to be involved in regulating the expression of iNOS and COX-2 (Surh et al., 2001). The most significant function in the NF-κB signaling pathway is performed by the transcription factor p65, which is a member of the NF-κB family. The activated NF-κB is translocated from the cytosol into the nucleus, and phosphorylated NF-κB induces the production of inflammatory cytokines, resulting in inflammation (Cai et al., 2024).

**FIGURE 5:**
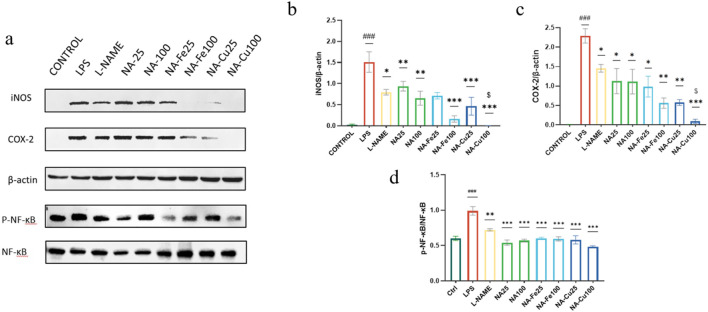
Effects of NA and its supramolecular complexes on the NF-κB/iNOS/COX-2 signaling pathway. The RAW264.7 cells were pretreated with 25 μg mL^−1^ and 100 μg mL^−1^ concentration of each drug for 2 h and stimulated with or without LPS (0.5 μg mL^−1^) for 18 h. The drugs and their different concentrations were analyzed by Western blotting **(a)**, and the expression of **(b)** iNOS, **(c)** COX-2, and **(d)** NF-κB p65 was assessed. The data are presented as mean ± standard deviation (n = 3); “*” compared to the LPS group, *p < 0.05, **p < 0.01, ***p < 0.001; “$” compared to the chlorogenic acids group, ^$^ *p* < 0.05; “#” compared to the control group, ^###^ *p* < 0.001.

In this study, the expression of the NF-κB pathway was detected by Western blotting. As indicated in Figure 5, the COX-2 and iNOS protein levels in the model group increased significantly (*p* < 0.001) and were significantly inhibited by NA and its supramolecules. In addition, LPS obviously induced phosphorylation of NF-κB p65 (*p* < 0.001), while the supramolecules switched this phenomenon. The results show that NA and its supramolecules could inhibit LPS induced by the NF-κB/COX-2/iNOS signaling pathway in a dose-dependent manner.

**FIGURE 6:**
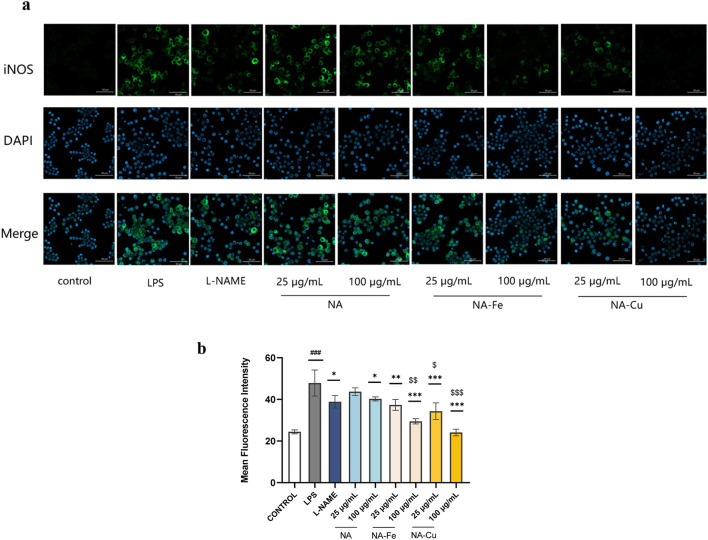
Immunofluorescence intensities of the expression of iNOS after treatment with NA and its supramolecular complexes **(a)** based on ImageJ measurements and **(b)** under different treatments compared to the LPS group. The data are presented as mean ± standard deviation (n = 3); “*” compared to the LPS group, *p < 0.05, **p < 0.01, ***p < 0.001; “$” compared to the chlorogenic acids group, ^$^ *p* < 0.05, ^$$^ *p* < 0.01, ^$$$^ *p* < 0.001; “#” compared to the control group, ^###^ *p* < 0.001.

The expression of iNOS was also validated through immunofluorescence staining. The LPS group showed significantly more green fluorescence than the control group, as illustrated in Figure 6, indicating greater expression of iNOS. Compared to the LPS group, treatment with NA, NA–Fe, and NA–Cu markedly inhibited NF-κB activation; in particular, the inhibition capability was better with NA–Cu than NA or NA–Fe.

### 3.6 Discussion

In this study, we successfully designed, synthesized, and characterized a new class of supramolecular complexes based on natural CGAs and metal ions (Fe^3+^ and Cu^2+^). Using a combination of UV-vis spectroscopy, FT-IR spectroscopy, and MS, we confirmed the formation of these complexes in a primarily 1:1 stoichiometry driven by metal-ligand coordination. Importantly, our work unambiguously demonstrates that this straightforward supramolecular strategy is a powerful means to significantly enhance the anti-inflammatory potency of the parent natural product. The anti-inflammatory efficacy of these CGA-metal supramolecules was rigorously evaluated in LPS-induced RAW264.7 macrophages. The results show that the supramolecules, particularly NA-Fe and NA-Cu, exhibit superior dose-dependent inhibition of the production of key inflammatory mediators, including NO, IL-6, IL-1β, and TNF-α, thereby outperforming their native counterpart NA. Further mechanistic investigations revealed that such enhanced activities were achieved through suppression of the NF-κB signaling pathway, which effectively inhibited phosphorylation of the NF-κB p65 subunit and subsequently downregulated the expression of downstream effector proteins iNOS and COX-2.

While the spectroscopic data provide compelling evidence of the formation of the supramolecules, we acknowledge the limitations of our current structural characterizations. The present data do not allow precise elucidation of the three-dimensional spatial structures at the atomic level or the exact aggregation morphologies in solution. Employing X-ray diffraction to determine the single-crystal structure or utilizing transmission electron microscopy and dynamic light scattering to characterize the nanoscale morphologies and particle size distributions will be critical steps in our future research. These in-depth characterizations are expected to provide a solid foundation for establishing the precise structure–activity relationships and are a central direction for our subsequent work.

Previous studies on CGA assemblies have largely focused on their applications as material building blocks and demonstrated the self-assembly of CGAs into hydrogels for wound dressing (Huang et al., 2023). In contrast, the core objective of our work is to not construct macroscopic materials but employ supramolecular chemistry as a “molecular engineering” strategy to create new pharmacological entities that can directly enhance the intrinsic efficacies of the natural small molecules. Although the antioxidant and antimicrobial activities of some metal-phenolic complexes have been reported, their action mechanisms are often broadly attributed to free-radical scavenging (Huang et al., 2022). Our pioneering study clearly demonstrates through robust molecular biological evidence that the CGA-metal supramolecules function by modulating the classic NF-κB/iNOS/COX-2 inflammatory signaling axis. This elevates the understood mechanism from a non-specific “antioxidant” activity to precise regulation of a specific signaling pathway, significantly enhancing our pharmacological understanding. This study not only confirms the formation of supramolecules but also quantitatively demonstrates a significant enhancement of the anti-inflammatory potential through systematic comparisons with the original drug (NA) and a clinical positive control, thus providing compelling experimental evidence for the development of supramolecular complexes as efficient anti-inflammatory lead compounds.

## 4 Conclusion

In this work, we developed and characterized supramolecules of CGAs with Fe and Cu. These supramolecular complexes are shown to have significant anti-inflammatory activities that exceed those of the original drugs as well as regulate the iNOS/COX-2/NF-κB signaling pathway. Our findings provide a basis for the design and synthesis of effective anti-inflammatory drugs. In the future, we believe that more natural molecules with self-assembly functions will continue to be developed in an effort to formulate safe and efficient drugs for anti-inflammatory research.

## Data Availability

The original contributions presented in the study are publicly available in Mendeley Data. This data can be found at: 10.17632/sdwn8x4kwg.1.
