# Supplementary figures and images for: Characterization and anti-inflammatory studies of supramolecular assemblies of chlorogenic acids with metal ions
Source: Front Pharmacol. 2026 Feb 9;16:1726226. doi: 10.3389/fphar.2025.1726226 (PMC12926475; doi:10.3389/fphar.2025.1726226)

0813

actin

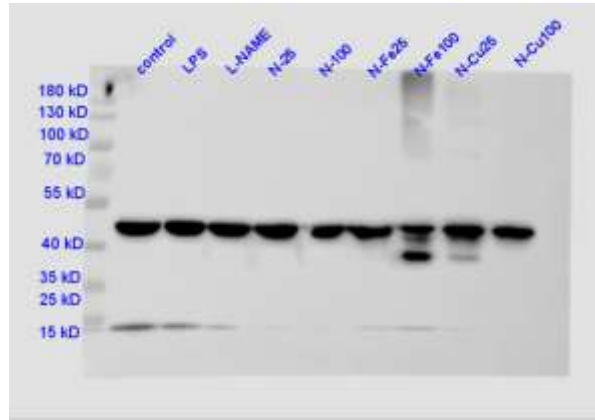

P-p65

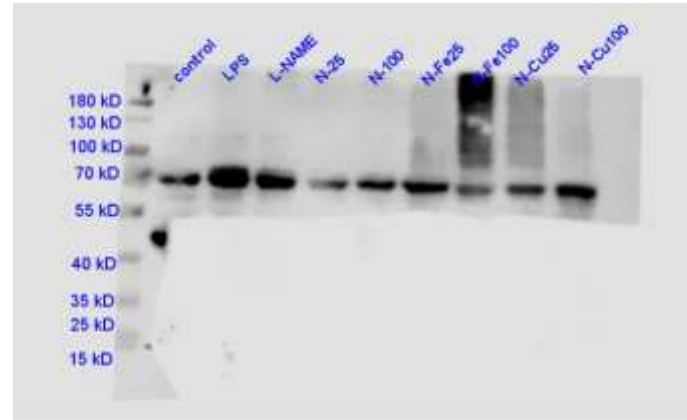

p65

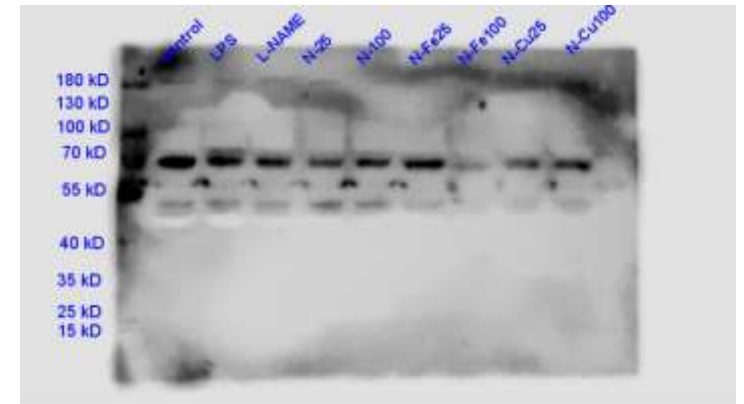

0812

actin

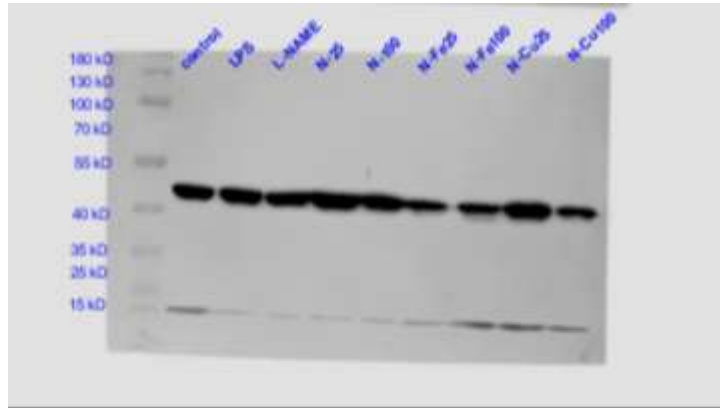

P-p65

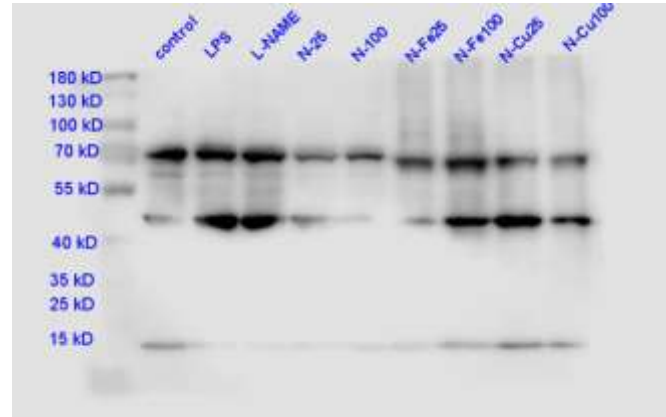

p65

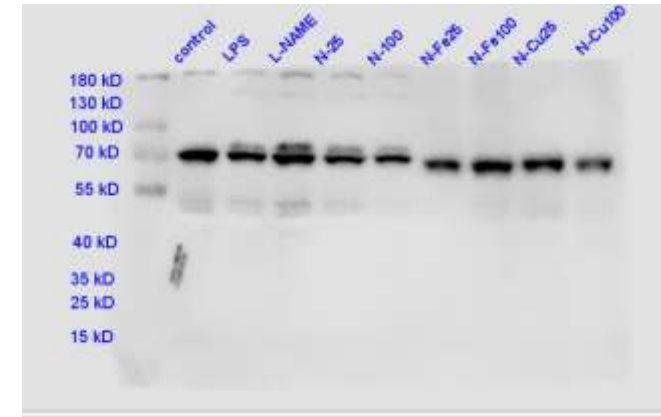

0811

actin

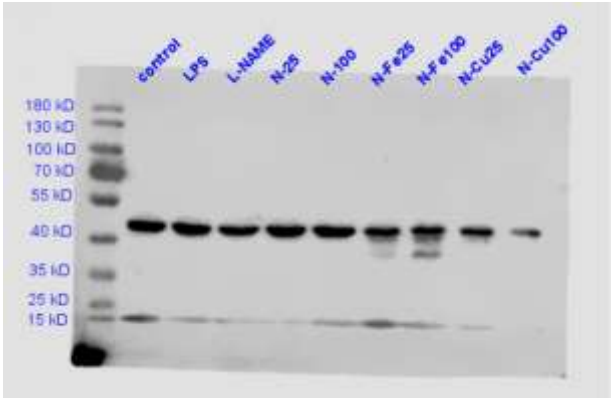

P-p65

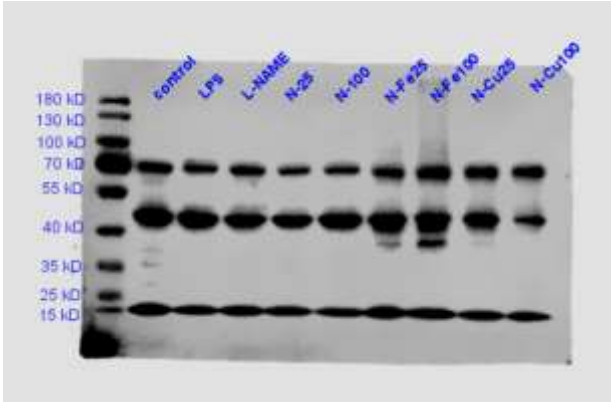

p65

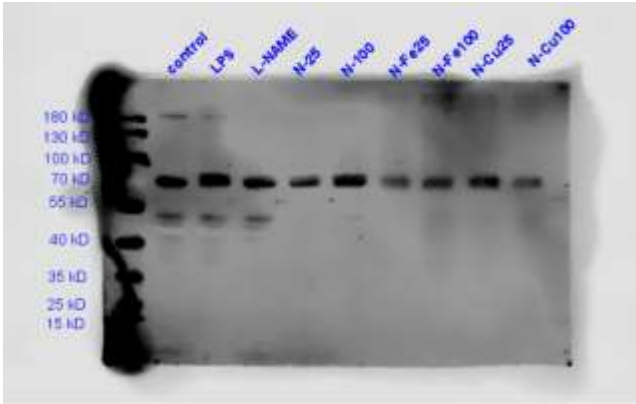

6092

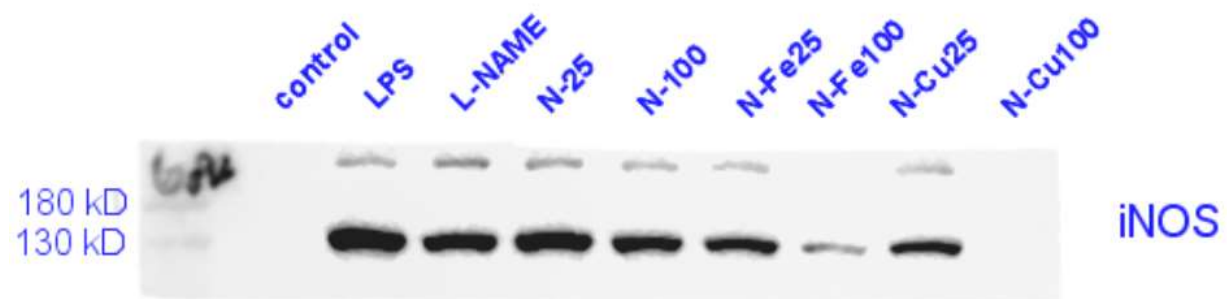

actin

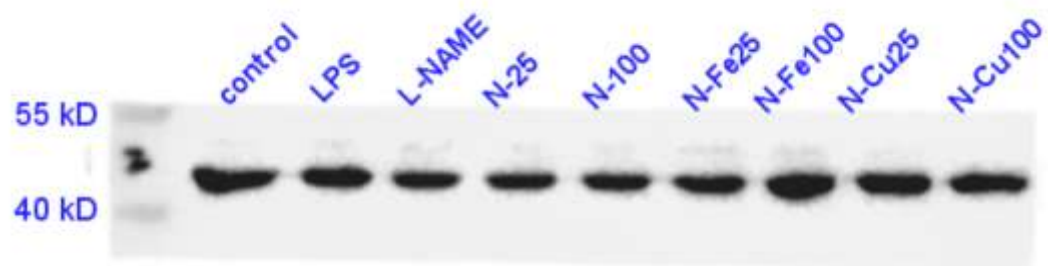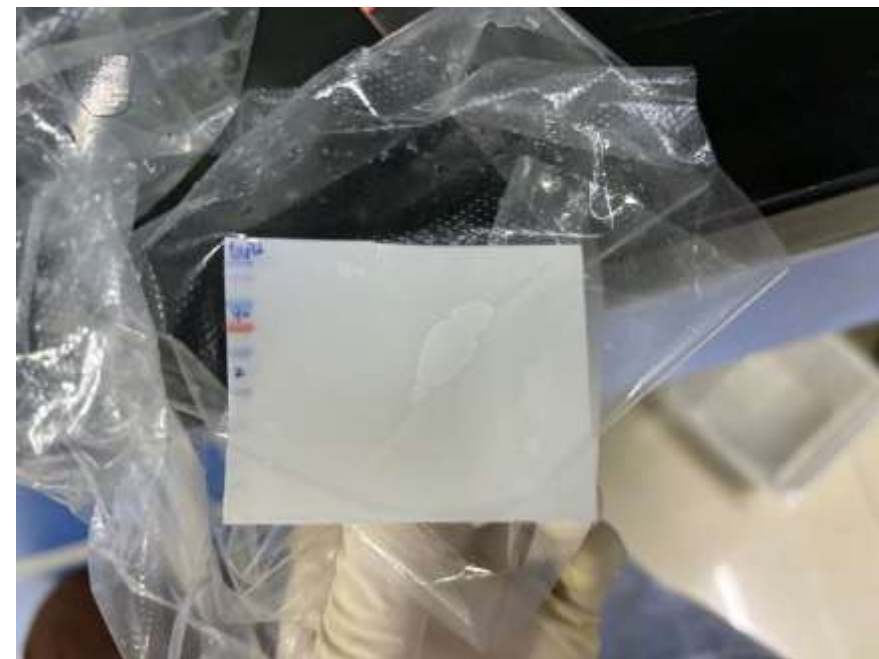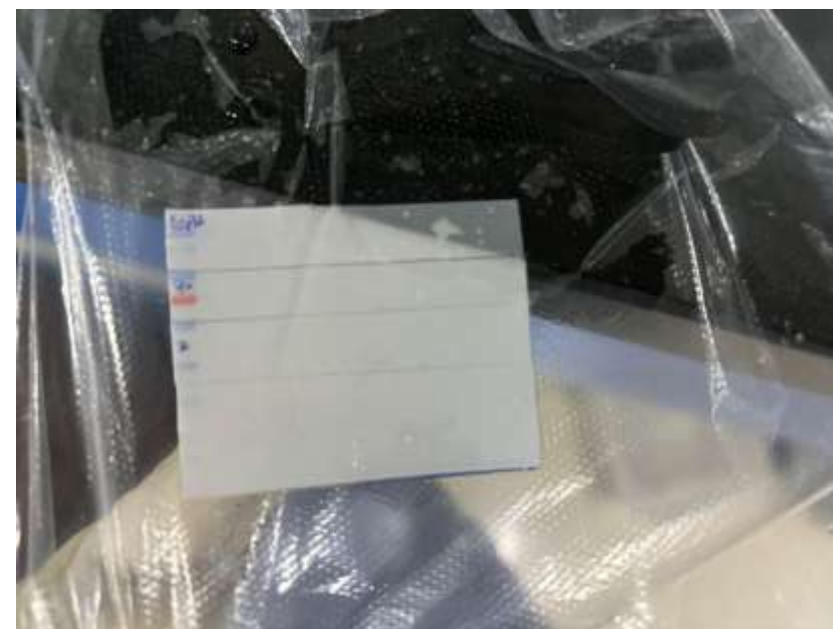

6072

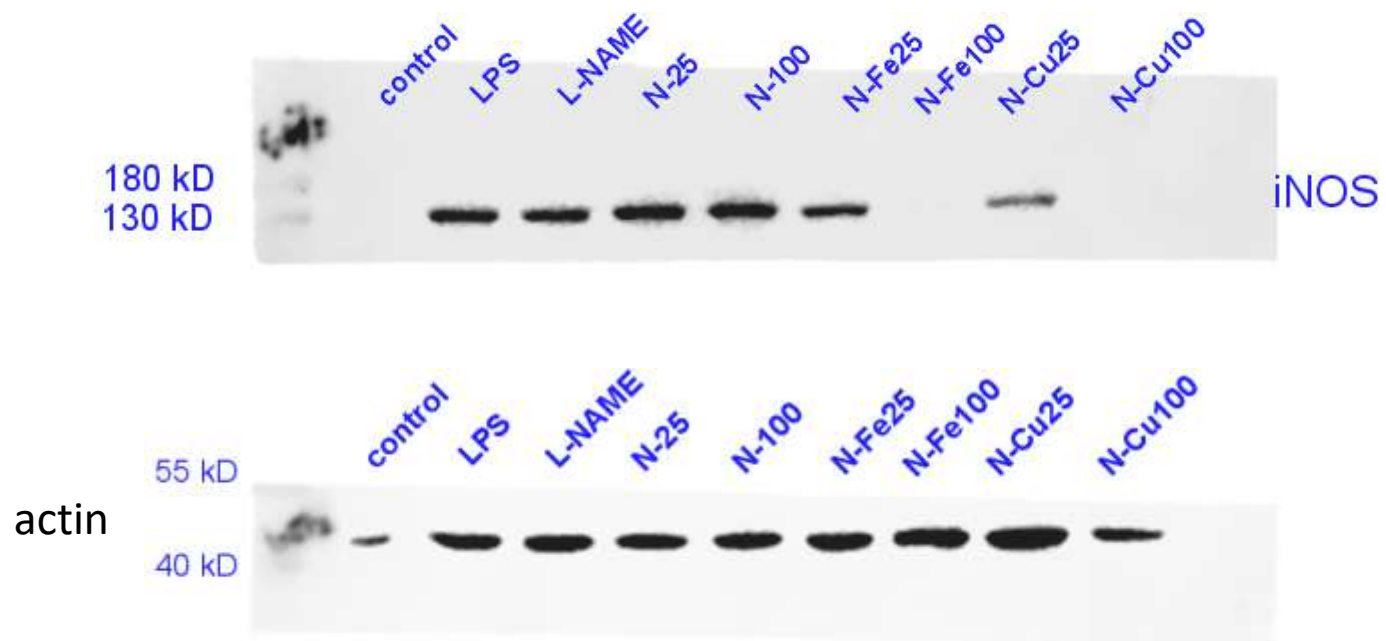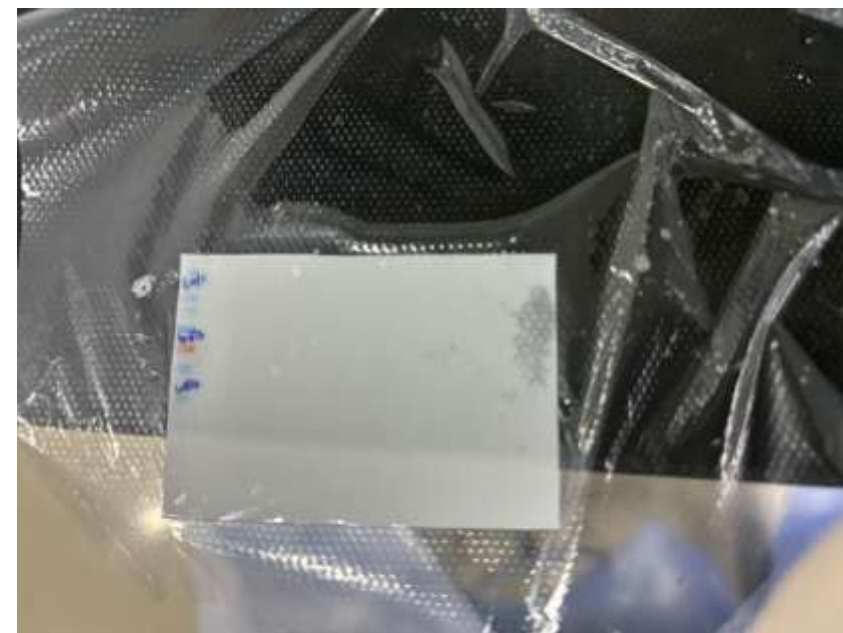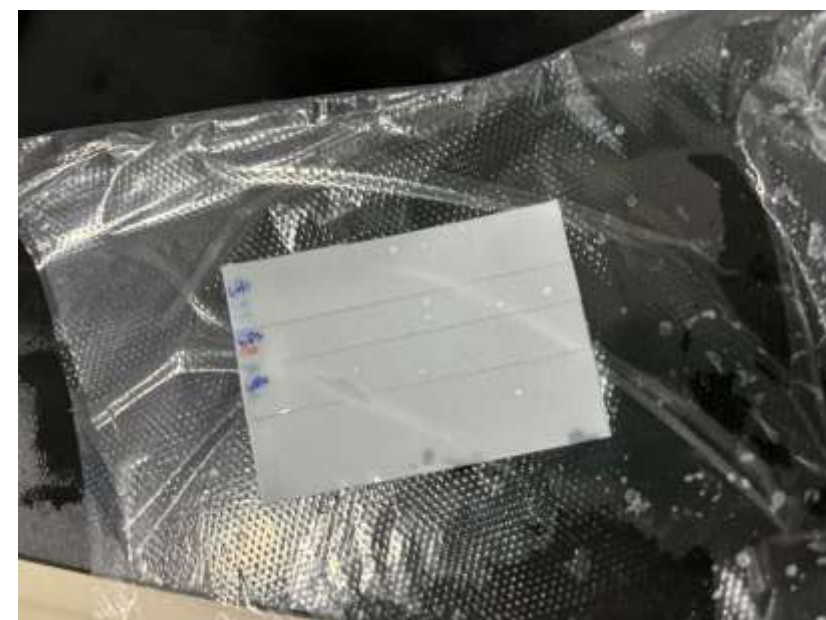

6071

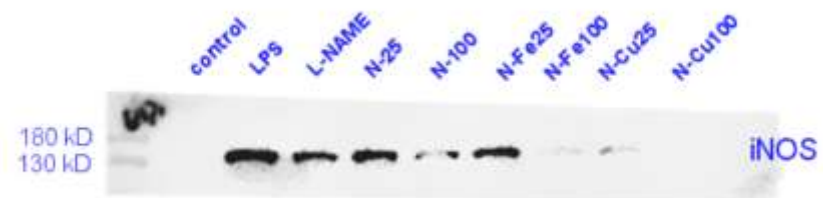

actin

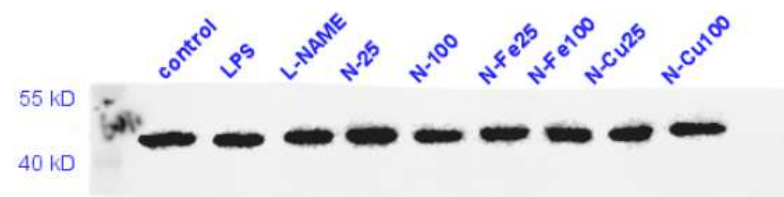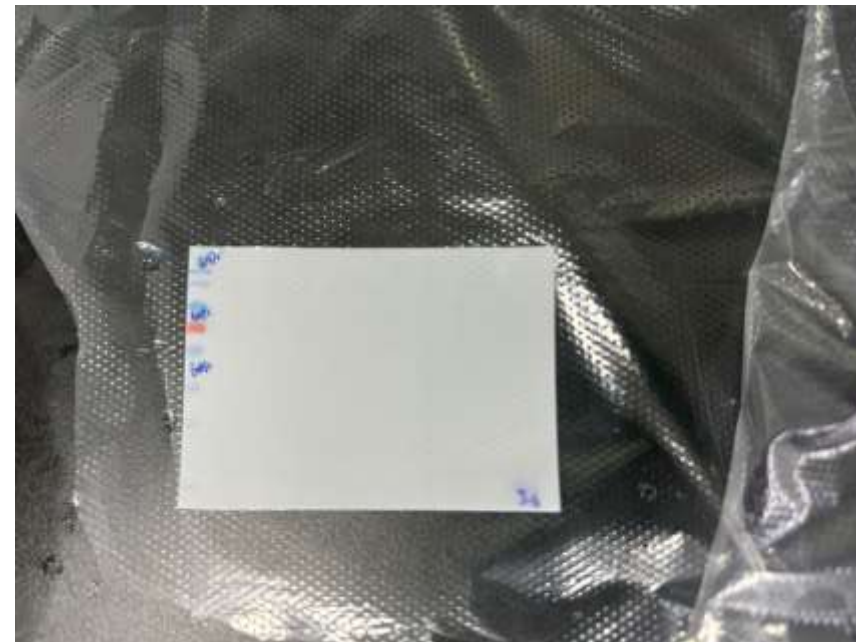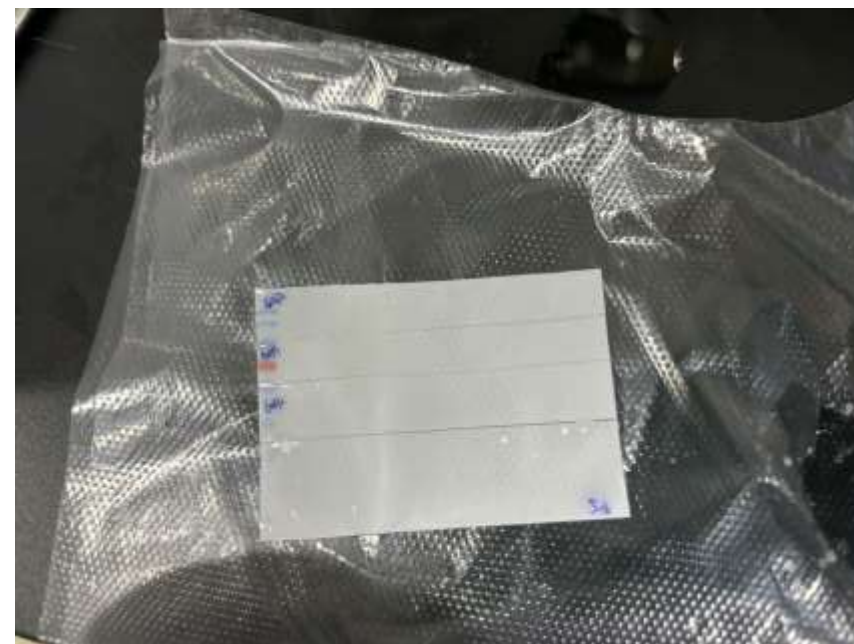

612

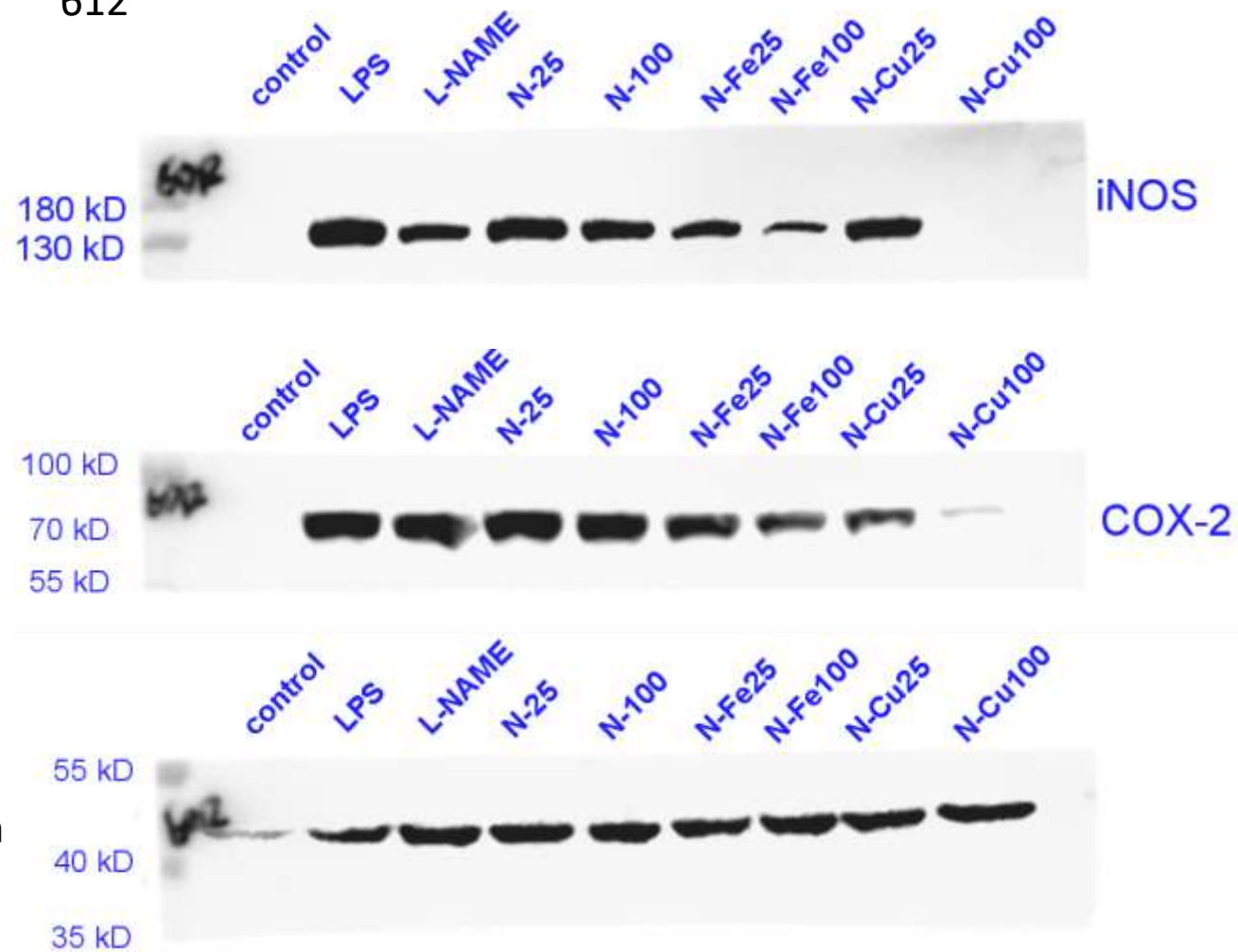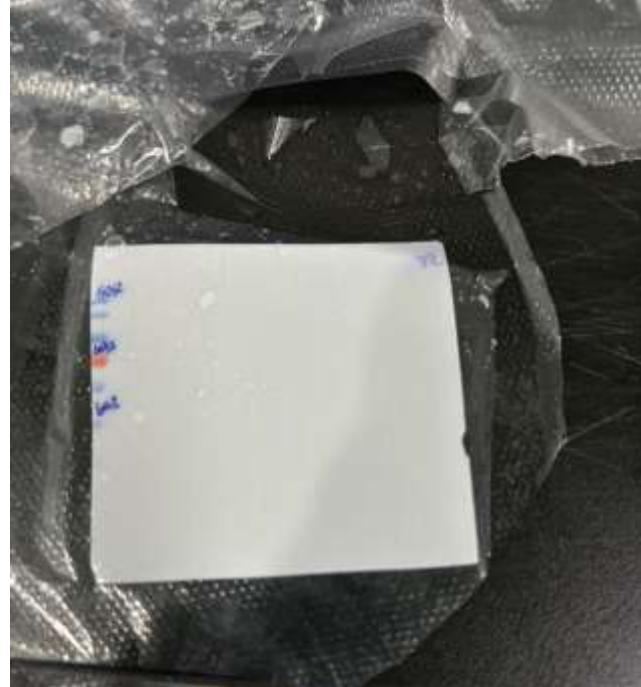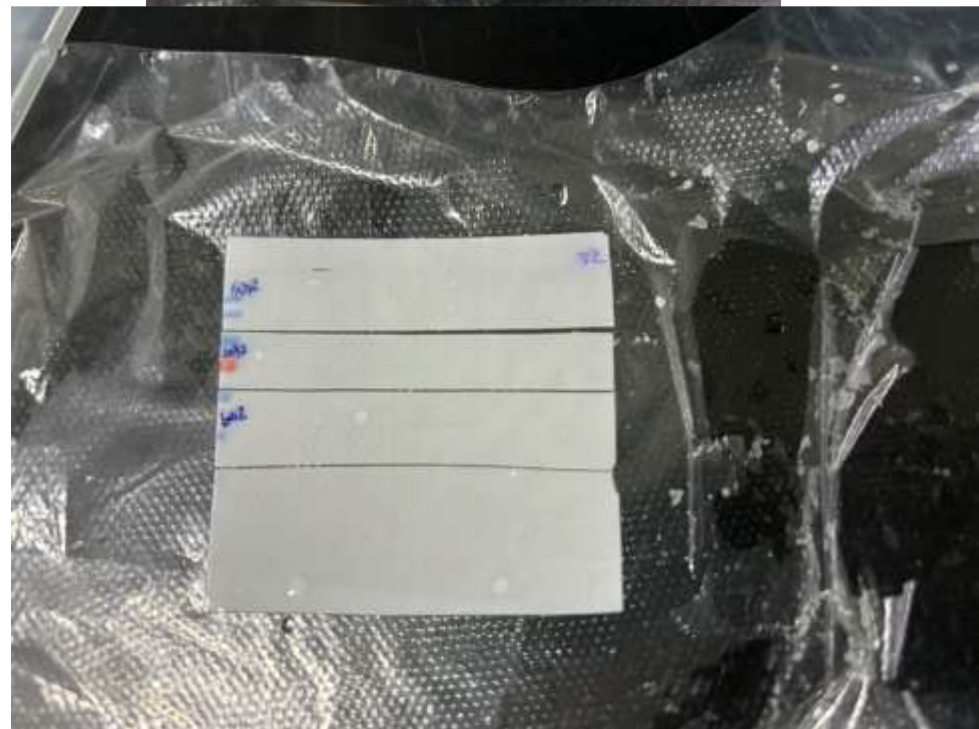

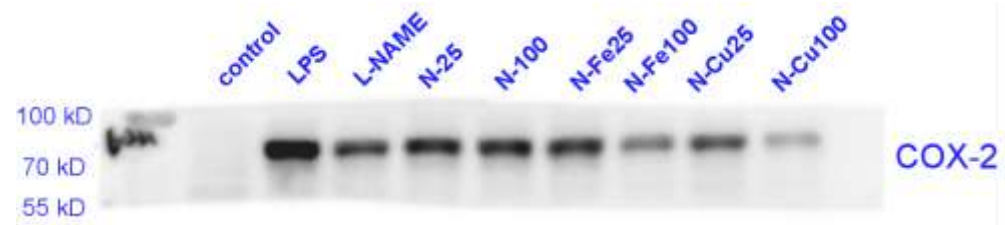

actin

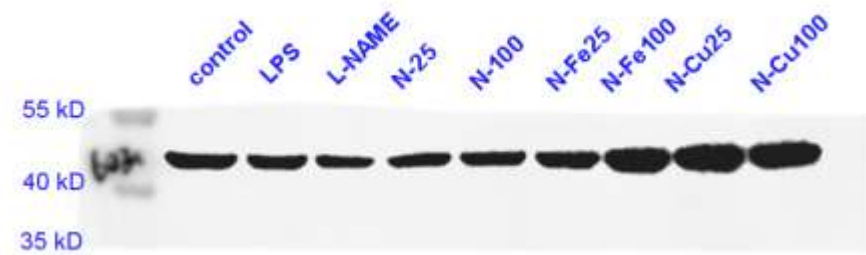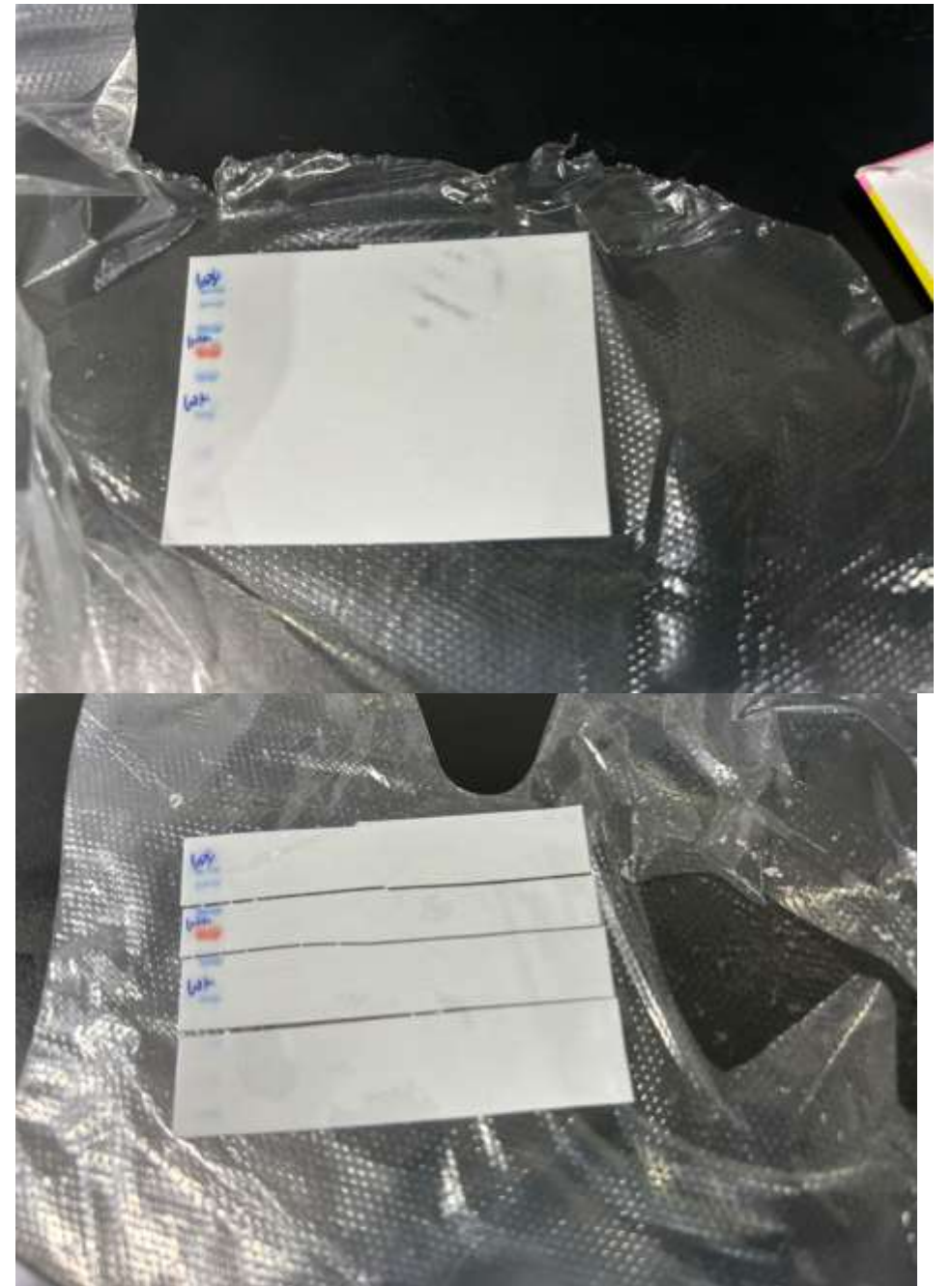

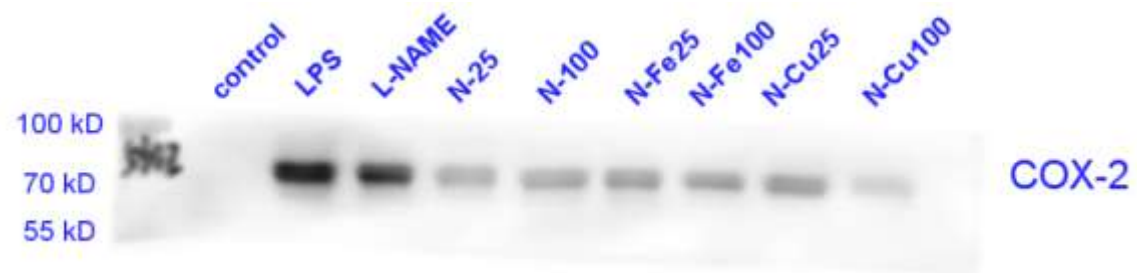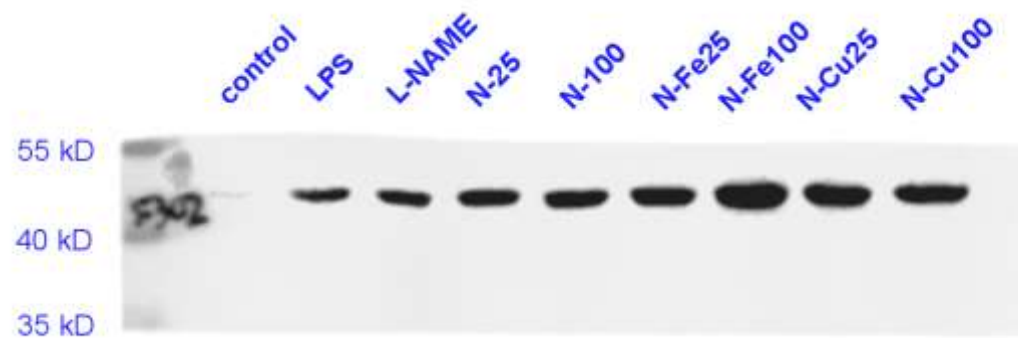

actin

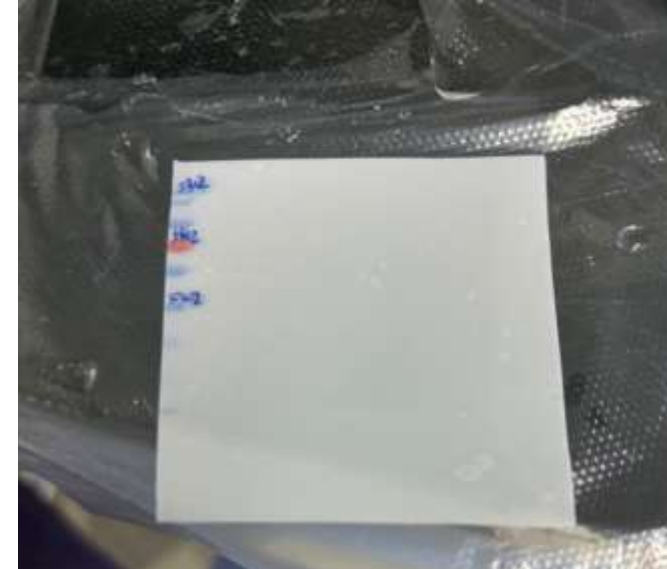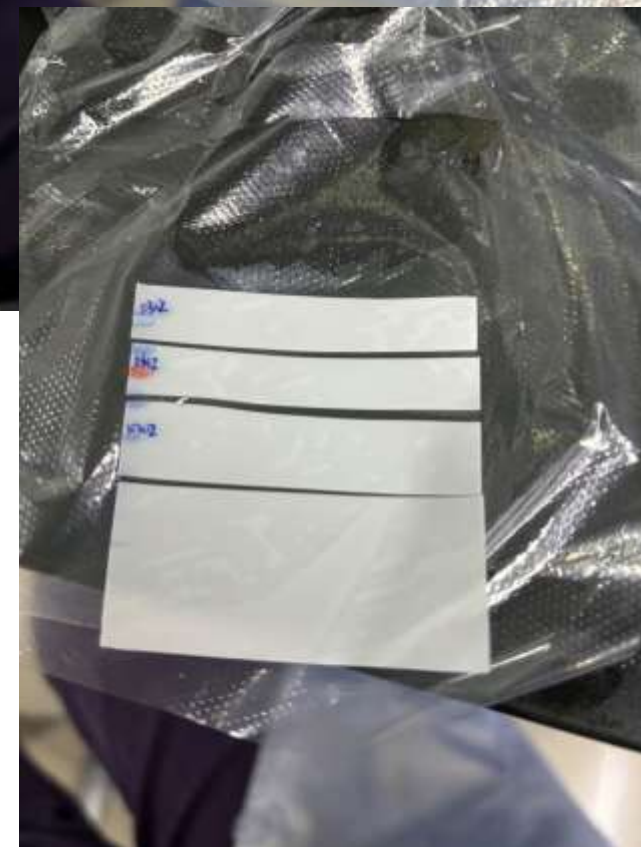

Supplement: Supplementary file 1 [file DataSheet1.PDF]
